# Systems biology approach highlights mechanistic differences between Crohn’s disease and ulcerative colitis

**DOI:** 10.1038/s41598-021-91124-3

**Published:** 2021-06-01

**Authors:** Pedro A. Ruiz Castro, Hasmik Yepiskoposyan, Sylvain Gubian, Florian Calvino-Martin, Ulrike Kogel, Kasper Renggli, Manuel C. Peitsch, Julia Hoeng, Marja Talikka

**Affiliations:** Philip Morris International R&D, Philip Morris Products S.A., Quai Jeanrenaud 5, 2000 Neuchâtel, Switzerland

**Keywords:** Cellular signalling networks, Computational models, Gastrointestinal models, Inflammatory bowel disease

## Abstract

The molecular mechanisms of IBD have been the subject of intensive exploration. We, therefore, assembled the available information into a suite of causal biological network models, which offer comprehensive visualization of the processes underlying IBD. Scientific text was curated by using Biological Expression Language (BEL) and compiled with OpenBEL 3.0.0. Network properties were analysed by Cytoscape. Network perturbation amplitudes were computed to score the network models with transcriptomic data from public data repositories. The IBD network model suite consists of three independent models that represent signalling pathways that contribute to IBD. In the “intestinal permeability” model, programmed cell death factors were downregulated in CD and upregulated in UC. In the “inflammation” model, PPARG, IL6, and IFN-associated pathways were prominent regulatory factors in both diseases. In the “wound healing” model, factors promoting wound healing were upregulated in CD and downregulated in UC. Scoring of publicly available transcriptomic datasets onto these network models demonstrated that the IBD models capture the perturbation in each dataset accurately. The IBD network model suite can provide better mechanistic insights of the transcriptional changes in IBD and constitutes a valuable tool in personalized medicine to further understand individual drug responses in IBD.

## Introduction

Inflammatory bowel disease (IBD), comprising Crohn’s disease (CD) and ulcerative colitis (UC), represents a group of chronic relapsing inflammatory disorders that affect the gastrointestinal tract. CD is defined as a segmental, transmural disease which can occur in any part of the gastrointestinal tract, whereas UC shows continuous involvement of the mucosal and submucosal layers of the rectum, moving up in varying degrees to the colon. IBD is thought to result from an aberrant inflammatory response to commensal microbes triggered by one or more environmental exposures in a genetically susceptible host, finally leading to chronic intestinal inflammation^1^.

There is increasing evidence that intestinal barrier function disturbance, coincident with deregulated immune response to enteric bacteria, plays a major role in the development of IBD^2^. The importance of the epithelial barrier in IBD predisposition is supported by reports of abnormal intestinal permeability in patients with IBD and some of their first-degree relatives^3–5^. Moreover, several lines of evidence support that a barrier defect alone can be sufficient for developing IBD^6^. The intestinal epithelium consists of a monolayer of nonciliated, columnar epithelial cells that separates the gut microbiota from the mucosal immune system present in the lamina propria^7^. Intestinal epithelial cells (IEC), which include absorptive epithelial cells, goblet cells, and Paneth cells, are primarily involved in absorption of water and nutrients while restricting the entry of luminal pathogens. Two main characteristics are key in maintaining epithelial barrier function: a tightly regulated balance between epithelial cell proliferation and cell death and effective sealing of the intercellular space by tight junction proteins. Alterations in the balance between proliferation and apoptosis are known to be involved in barrier dysfunction, which leads to IBD^8^. This is further supported by evidence showing that anti-tumor necrosis factor (TNF) therapies reduce IEC apoptosis in models of colitis and CD patients^9,10^.

Tight junctions are multiprotein complexes formed by integral proteins, including occludin, zonula occludens (ZO), and claudins^11^. TNF has been shown to regulate the architecture of tight junctions altering the expression and localization of tight junction proteins, including ZO-1, and promoting occludin endocytosis^12,13^. Defensins secreted by Paneth cells play a key role in host defense against the invasion of microorganisms, and their expression has been shown to be altered in IBD^14^. Additionally, the pathways of several genetic risk factors for IBD impair barrier function and lead to colitis, including the NOD2 (nucleotide-binding oligomerization domain containing 2) and autophagy pathways. NOD2 risk variants are linked to lower expression of α-defensins in Paneth cells, leading to impaired responses against invading microorganisms^15^. Paneth cell function is also compromised in CD patients through the risk autophagic gene ATG16L1^16,17^.

IECs express various pattern recognition receptors such as Toll-like receptors (TLR) that are able to recognize conserved microbial components (e.g. lipopolysaccharides)^18^. In response to these signals, IECs produce a variety of inflammatory cytokines, such as interleukin (IL) 8^19^, CXCL (C-X-C motif chemokine ligand) 2^20^, and C–C motif chemokine ligand 20^21^. Patients with CD have deficient innate immune responses, including reduced macrophage activity and neutrophil recruitment, enabling microbes to traverse the intestinal mucosa. In response to pathogen- and danger-associated molecular patterns, inflammasome sensors—including NLRP (nucleotide-binding oligomerization domain, leucine rich repeat [NLR] and pyrin domain containing protein) 1, NLRP3, NLRP6, NLRC (NLR family CARD domain containing) 4, NAIP (neuronal apoptosis inhibitory protein), and AIM (absent in melanoma) 2—associate with the adaptor protein PYCARD (often referred to as ASC), which results in the activation of CASP1 (cysteine protease caspase 1) and the subsequent proteolytic processing of IL1β and IL18^22^. NLRP3 is one of the best characterized inflammasome sensors and a key mediator of intestinal inflammation in vivo^23,24^. Importantly, single nucleotide polymorphisms in the *Nlrp3* gene have been associated with the development of CD^25^. IL1β is highly produced in the colon of IBD patients and promotes the activation of dendritic cells, macrophages, and neutrophils^23^.

Whereas CD results from an excessive Th1 (type 1 helper T) and Th17 cell response, UC is driven by a Th2 cell-type-like cytokine profile^26^. In CD, the differentiation of Th1 and Th17 occurs in response to IL12, IL18, IL23, and TGF (transforming growth factor) β1. In turn, Th1 and Th17 cells secrete the proinflammatory cytokines IL17, interferon (IFN) γ, and TNF, which stimulate the production of TNF, IL1, IL6, IL8, IL12, and IL18 in antigen-presenting cells, macrophages, fibroblasts, and endothelial cells^27^. The abundance of TGFβ1 in intestinal tissues contributes to normal homeostasis by promoting Treg differentiation in naive lamina propria CD4 + T cells^28^.

Rapid resealing of the epithelial surface barrier following physiological damage is essential for re-establishing intestinal homeostasis^29^. An appropriately resolving inflammatory process results in successful wound healing, while a sustained, nonresolving inflammation is often related to an overactive wound healing response, leading to tissue fibrosis, a major clinical complication in IBD. Approximately one-third of patients with CD require surgical resection of fibrotic tissue in the colon^30^. The wound healing response involves a series of seamless and overlapping inflammatory, proliferative, and remodelling phases, with each phase requiring the participation of specific cell types (including immune, mesenchymal, and IECs) and mediators^31^. During the inflammatory phase, resident cells produce cytokines, which recruit immune cells to the site of injury. Infiltrating monocytes differentiate into macrophages, key players in driving an effective immune response through phagocytosis of pathogens and apoptotic neutrophils^32^. Mediators released during the inflammatory phase also recruit fibroblasts to the wound region, thereby initiating the proliferative phase, characterized by the deposition of extracellular matrix (ECM) proteins such as fibronectin and collagens^33^. TGFβ1 is regarded as the master regulator of wound healing and fibrosis processes^34^. TGFβ1 triggers the proliferation and differentiation of fibroblasts and is a potent inducer of major ECM proteins^35^. TGFβ signalling also promotes epithelial–mesenchymal transition (EMT)^36^.

Review articles on IBD generally focus on specific signalling aspects, and the rest of the disease mechanistic information is scattered in a myriad of publications, leaving a lot of the information uncaptured in a formalized way. Hence, to efficiently utilize the available information and identify information gaps, there is a need to collate the scientific findings into an easy, browsable, visual representation. To this end, the scientific findings can be scripted into cause-and-effect relationships by using the computable language Biological Expression Language (BEL) and compiled into causal biological network (CBN) models. Over the years, we have built several CBN models that capture biological processes, mainly in the pulmonary and vascular context^37^. As shown previously, CBN models are computable and can be used to interpret transcriptomic data. Initially designed for toxicological studies^38,39^, these models could be employed in several applications, such as disease mechanism studies^40^, drug discovery^41^, drug safety studies, and personalized medicine^42^. The models are stored in the CBN database for browsing and download for public use^37,41,43,44^.

In this study, we present a new addition to the CBN, a suite of causal biological models that describe important molecular events involved in IBD, from epithelial barrier defence to inflammation and wound healing. We score transcriptomic data from the colon of CD and UC subjects onto the merged IBD network model to show how the model can provide mechanistic understanding from gene expression changes in diseased tissue.

## Materials and methods

### Biocuration and network model assembly

The first step in network model building was to identify scientific literature on the known signalling pathways involved in barrier defence, inflammatory processes, and wound healing in IBD. We chose only articles with mechanistic information, i.e., demonstrated causal relationships between biomolecules, and captured them in a computable format by using the Biological Expression Language (BEL) version 1.0, which converts the relationships between biomolecules into cause-and-effect statements by using controlled vocabularies, which facilitate subsequent computation of the models. Each BEL statement consists of a source, relationship, and target, where the source and target are biological entities that are defined by a BEL function (RNA abundances, protein abundances, protein activities, protein families, complexes, cellular structures, chemical abundancies, biological processes, and pathologies), and the entity name is scripted in a specific namespace, such as the HGNC (HUGO Gene Nomenclature Committee) for human proteins^45^. When a biological entity was not available in any of the existing namespaces, we created a term in a custom namespace (e.g., PMICOMP for complex). As BEL allows us to script mRNAs, protein abundances, and activities (BEL functions) for a given gene product, we followed certain rules. When a gene product appeared as a source node (i.e., causing something), we scripted it as protein activity, except in case of components of cellular structures and microRNAs. When a gene product appeared as the target of a statement, we assigned the BEL function depending on the assay that was used for detection: qRT-PCR → mRNA, western blot → protein abundance, activity assay → protein activity.

We also captured as much contextual detail as possible (e.g., species, cell/tissue type, and disease state) as annotations with each BEL statement. The curated articles were studies in mouse and rat IBD models as well as human, mouse, rat, and canine cell lines. After curating the evidence from these articles, we orthologized network models to HGNC nomenclature and compiled all BEL statements into network models by using the OpenBEL framework 3.0.0 (https://github.com/OpenBEL/openbel-framework). We reviewed the network models using the Cytoscape web application to identify any gaps and then performed additional curation. Finally, we refined and trimmed the models to avoid too much overlap between the individual models and eliminate any entities that were “dead ends” that could not be linked to the biological processes or diseases described in the model even after additional extensive literature search.

### Model analysis

We used the Cytoscape analysis tool to compute node degree distribution and identify the most connected nodes for each IBD network model by using statistics for directed network^46^.

### Transcriptomic data analysis

**Figure 1:**
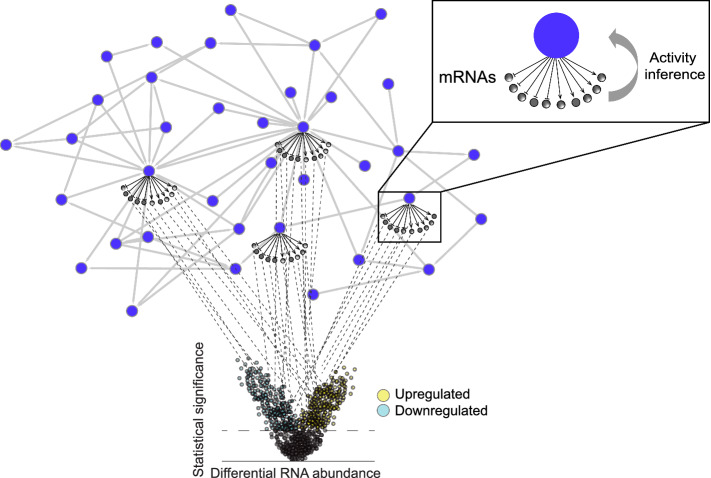
Node scoring with transcriptomic data. In addition to the backbone layer, which describes the molecular pathways involved in each biological process, the network models have a second layer, which harbours information about mRNA regulated by some of the entities in the model backbone (inset above). The activity of the nodes in the model backbone is inferred on the basis of the concordance of transcriptomic changes in the data with the mRNA nodes underneath the backbone nodes. The figure was created using Cytoscape v3.7.1 (https://cytoscape.org). The Cytoscape graph was then exported to pdf and the layout was further modified for better readability in Adobe Illustrator 2019 (https://adobe.com).

To allow model scoring, we added information about RNA regulation to as many network nodes as possible. Such nodes with regulated mRNAs underneath are called iNodes (inferable), the activity of which can be inferred from gene expression fold changes in the data (Fig. 1). Some of the RNA nodes were obtained during literature curation, and, to create more iNodes, we used public datasets with significant gene expression changes in response to manipulation (knockout, dominant negative, overexpression, chemical inhibition, etc.) of the nodes of interest. We downloaded the processed transcriptomic data from the Genvestigator platform (https://genevestigator.com/), and, to score the model nodes, we used the Strength network perturbation amplitude scoring algorithm, which is a threshold-free enrichment method specific for iNode that accounts for the direction of regulation of mRNAs, as described in detail by Martin et al*.*^47^. To graphically illustrate how each node was inferred on the basis of gene expression differences in the dataset, we imported the inference values to Cytoscape^46^ and applied them to model nodes.

## Results

### Network model description

This study presents a detailed description of the biomolecules and pathways that are involved in the IBD pathology by bridging literature-extracted IBD mechanistic knowledge with IBD patient-derived transcriptomic data. The information is organized in network models focused on epithelial barrier defence, inflammation, and wound healing processes. The inflammation network model illustrates various pathways involved in the onset, persistence, and management of inflammation in IBD. The barrier defence network model visualizes the routes that lead to tight junction disruption and highlights the molecular entities involved in the balance between intestinal epithelial cell proliferation and apoptosis, which is essential for tight junction regulation. The wound healing network model addresses the repair mechanisms of tissue damage caused by acute and chronic intestinal inflammation. This network model explores the pathways that lead to epithelial cell proliferation, deposition of extracellular matrix proteins, fibroblast migration, and tissue remodelling. In the models, the biological entities are represented as nodes that are connected by edges, which define the relationships between the nodes. The model contains causal relationships between active proteins, protein complexes, and regulatory microRNAs and their targets, and the signalling pathways composed of these components lead to biological processes and pathologies, when applicable. The figure was created using Cytoscape v3.7.1 (https://cytoscape.org). The Cytoscape graph was then exported to pdf and the layout was further modified for better readability in Adobe Illustrator 2019 (https://adobe.com).

Table 1 shows the number of scientific articles curated as well as the number of nodes and edges in each model, and Table 2 lists the most connected nodes in each model. The network models are available for browsing and download in the CBN database (http://causalbionet.com/).

**Table 1:** Network model statistics.

Model	Scientific articles (publication years)	Nodes	Causal edges
Barrier defense	64 (2001–2019)	281	502
Inflammation	80 (1993–2020)	354	500
Wound healing	33 (1996–2019)	176	200

**Table 2:** The most connected nodes.

Node name
**Barrier defense**
bp(PMIBP:"tight junction disruption")
complex(GOCC:"NF-kappaB complex")
act(p(SFAM:"TNFRSF Family"))
bp(PMIBP:"Intestinal permeability")
bp(GOBP:"programmed cell death")
p(HGNC:OCLN)
complex(SCOMP:"Interferon Gamma Receptor Complex")
act(p(HGNC:MYLK))
act(p(HGNC:VDR))
act(p(SFAM:"MAPK JNK Family"))
**Inflammation**
path(MESHD:Inflammation)
complex(GOCC:"NF-kappaB complex")
complex(GOCC:"interleukin-1 receptor complex")
act(p(HGNC:MEFV))
act(p(HGNC:MYD88))
act(p(HGNC:STAT3))
act(p(HGNC:NOD2))
act(p(HGNC:MMP19))
act(p(HGNC:PPARG))
p(HGNC:IL6)
**Wound healing**
bp(GOBP:"cell proliferation")
bp(GOBP:"epithelial cell migration")
bp(GOBP:"wound healing")
complex(GOCC:"NF-kappaB complex")
act(p(HGNC:CTNNB1))
act(p(HGNC:CRHR2))
act(p(SFAM:"TNFRSF Family"))
complex(PMICOMP:"FGF receptor complex")
act(p(HGNC:MMP9))
act(p(HGNC:STAT3))

### Interpretation of transcriptomic data by using the IBD network model

To demonstrate the utility of the IBD network model suite for mechanistic understanding of the disease, we used E-MEXP-2083 gene expression data, created from colon mucosa samples that showed the typical histological parameters of active CD or UC^48^. Briefly, the differential mRNA abundances in affected and control tissues were scored against the iNodes, and the results of the scoring show if the respective iNode is inferred to be activated or inhibited (Fig. 1).

### Nuclear factor (NF)-κB network model

**Figure 2:**
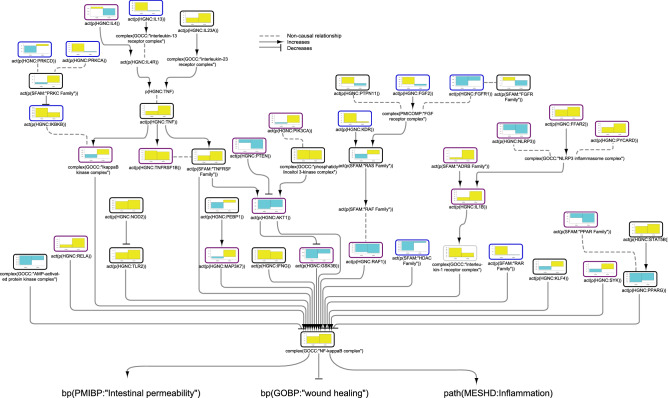
Impact of IBD on signalling pathways leading to NF-κB activation in colonic mucosa. The subgraph extracted from the merged IBD network model shows the iNode enrichment score for each node when scored with comparative transcriptomic data from IBD-affected versus healthy control colon samples. The values are not comparable between the graphs, and the scale is adjusted on the basis of the highest value for each node. Most intermediate nodes that were not scored have been removed for simplicity (The complete results are in Supplementary file 1). Black outlines indicate nodes that were significantly impacted in both CD and UC samples; blue outlines indicate nodes that were significantly impacted in CD samples; and purple outlines indicate nodes that were significantly impacted only in UC samples. The directionalities are shown as yellow or turquoise bars for inferred upregulation or downregulation, respectively. The order is as follows: 1) CD and 2) UC. Abbreviations: act, protein activity; p, protein abundance; bp, biological process. HGNC is the namespace for human proteins; GOBP is the namespace for biological processes; MESHD is the namespace for diseases; and GOCC is the namespace for complexes. SFAM and PMIBP are custom namespaces for protein families and biological processes, respectively. The figure was created using Cytoscape v3.7.1 (https://cytoscape.org). The Cytoscape graph was then exported to pdf and the layout was further modified for better readability in Adobe Illustrator 2019 (https://adobe.com).

As NF-κB is one of the key regulators of the processes described in our network model suite, we first investigated the impacted pathways that led to NF-κB activation/inhibition, as shown in Fig. 2. The iNode scoring algorithm inferred upregulation of NOD2, TLR2, and NF-κB in both CD and UC colon samples; however, some of the pathways upstream of NF-κB showed divergent regulation in CD and UC samples. FFAR2 (free fatty acid receptor 2) was inferred to be significantly upregulated only in UC colon. FFAR2 activates the NLRP3 inflammasome complex, which is composed of PYCARD and NLRP3. Interestingly, while FFAR2 and PYCARD were inferred to be upregulated, NLRP3 was inferred to be downregulated in UC colon samples. Inflammasome-associated CASP1 was not inferred to be significantly regulated in either CD or UC (Supplementary file 1). In contrast, IL1β, which is cleaved to its active form by CASP1, was inferred to be activated in UC samples. The ADRB (β-adrenoreceptor) family, also upstream of IL1β in the model, was inferred to be activated in UC. Members of the PRKC (protein serine/threonine kinase) family were inferred to be downregulated in CD and upregulated in UC colon, and two members of this family, PRKCA and PRKCD, were inferred to be regulated in opposite directions in CD colon. The PRKC family negatively regulates IKKβ (inhibitor of nuclear factor kappa-B kinase subunit beta), whose activity was inferred to be upregulated in CD samples. The RAR (retinoic acid receptor) family and HDAC (histone deacetylases), negative regulators of NF-κB, were inferred to be regulated up and down, respectively, in CD. PEBP1 (phosphatidylethanolamine binding protein 1) was inferred to be downregulated in CD and upregulated in UC, and its downstream target, MAP3K7 (mitogen-activated protein kinase kinase kinase 7), was inferred to be upregulated only in UC colon. KLF (Krüppel-like factor) and SYK (spleen associated tyrosine kinase), positive regulators of NF-κB, were inferred to be upregulated in UC, whereas KLF was inferred to be downregulated in CD colon.

In addition to the divergent regulation of signalling in CD and UC samples, we discovered several causal inconsistencies in the inferred regulation of the model nodes. While IL13 was inferred to be upregulated only in CD and IL4 was inferred to be downregulated only in UC colon, TNF signalling was inferred to be upregulated in both CD and UC. AKT1 (RAC-alpha serine/threonine-protein kinase)—which receives positive regulation from TNF signalling and PI3K (phosphoinositide 3-kinase), both inferred to be upregulated—was inferred to be downregulated in UC colon samples. Moreover, its upstream suppressor, PTEN (phosphatase and tensin homolog), and downstream target, GSK3B (glycogen synthase-kinase 3B) were inferred to be downregulated, demonstrating causal inconsistency in the regulation of the nodes in the model. FGFR (fibroblast growth factor receptor) complex activity is impacted by its components as well as by positive regulation of FGF2, inferred to be upregulated in CD colon. The FGFR1 node was inferred to be downregulated in CD, and PTPN11 (protein tyrosine phosphatase non-receptor type 11) and the FGFR family showed opposite inferred regulation in CD and UC samples. The downstream target of the FGFR complex, the RAS family node, was inferred to be downregulated in CD and upregulated in UC, and RAF1 (rapidly accelerated fibrosarcoma proto-oncogene serine/threonine-protein kinase) was inferred to be downregulated in both CD and UC, but the downregulation reached statistical significance only in UC. Finally, the results of the iNode inference captured the inhibitory effect of STAT5B (signal transducer and activator of transcription 5B) on PPARG (peroxisome proliferator activated receptor gamma) activation, as PPARG was inferred to be downregulated, while the upstream regulator STAT5B, was inferred to be upregulated in both CD and UC.

### Intestinal permeability network model

Next, we investigated the pathways that lead to the three biological processes described in the model independent of NF-κB activation. To note, the model describes the interactions that were specifically shown in the IBD context. The effects of some of the molecules described below may be mediated via NF-κB in other tissue contexts and have not yet been experimentally confirmed in IBD. The figure was created using Cytoscape v3.7.1 (https://cytoscape.org). The Cytoscape graph was then exported to pdf and the layout was further modified for better readability in Adobe Illustrator 2019 (https://adobe.com).

**Figure 3:**
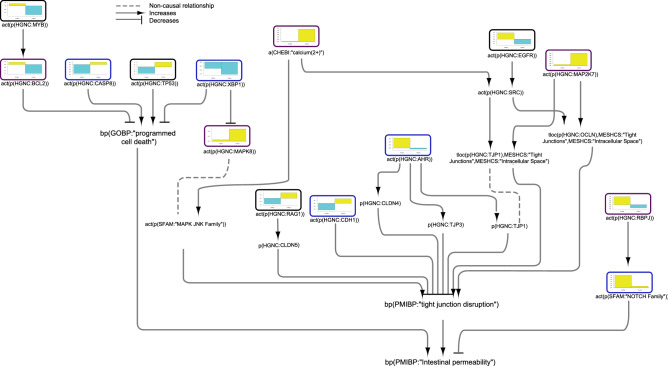
Impact of IBD on signalling pathways leading to intestinal permeability in colonic mucosa. The subgraph extracted from the merged IBD network model shows the iNode enrichment score for each node when scored with comparative transcriptomic data from IBD-affected versus healthy control samples. The values are not comparable between the graphs, and the scale is adjusted on the basis of the highest value for each node. Most intermediate nodes that were not scored have been removed for simplicity (The complete results are in Supplementary file 1). Black outlines indicate nodes that were significantly impacted in both CD and UC samples; blue outlines indicate nodes that were significantly impacted in CD samples; and purple outlines indicate nodes that were significantly impacted only in UC samples. The directionalities are shown as yellow or turquoise bars for inferred upregulation or downregulation, respectively. The order is as follows: 1) CD colon and 2) UC colon. Abbreviations: act, protein activity; p, protein abundance; bp, biological process. HGNC is the namespace for human proteins; GOBP is the namespace for biological processes; MESHD is the namespace for diseases; and GOCC is the namespace for complexes. SFAM and PMIBP are custom namespaces for protein families and biological processes, respectively. The figure was created using Cytoscape v3.7.1 (https://cytoscape.org). The Cytoscape graph was then exported to pdf and the layout was further modified for better readability in Adobe Illustrator 2019 (https://adobe.com).

Figure 3 shows the inferred regulation of signalling molecules that impact intestinal permeability in IBD. On the basis of the inferred regulation of CASP8, TP53, MYB, BCL2 (B-cell lymphoma 2), and XBP1 (X-box binding protein 1), programmed cell death appeared to be downregulated in CD and upregulated in UC colon. AHR (aryl hydrocarbon receptor)—known to increase the levels of TJP (tight junction protein) 1 and 3 as well as CLDN (claudin) 4—was inferred to be upregulated in CD colon, indicating a protective effect on tight junctions. JNK (Jun kinase), mitogen-activated protein kinase (MAPK) 8, and calcium, a known activator of JNK, were inferred to be upregulated in UC colon, promoting tight junction disruption in the model. In addition to calcium, EGFR (epidermal growth factor) activates SRC in the model and was inferred to be upregulated in CD and downregulated in UC colon. SRC and MAPK7 increase the translocation of OCLN (occludin) and TJP1 from tight junctions to the extracellular space, leading to tight junction disruption. The inferred upregulation of NOTCH further indicated that intestinal permeability was reduced in CD colon. However, RAG1 (recombination activating gene 1) and cadherin (CDH) 1, which protect the junctions, were also inferred to be downregulated in CD colon.

### Inflammation network model

**Figure 4:**
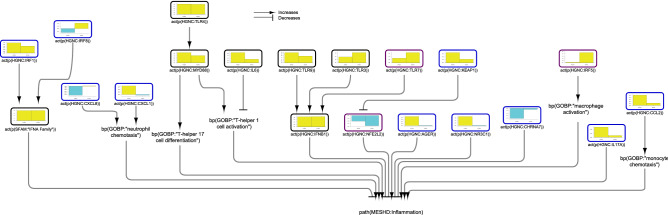
Impact of IBD on signalling pathways leading to inflammation in colonic mucosa. The subgraph extracted from the merged IBD network model shows the iNode enrichment score for each node when scored with comparative transcriptomic data from IBD-affected versus healthy control samples. The values are not comparable between the graphs, and the scale is adjusted on the basis of the highest value for each node. Most intermediate nodes that were not scored have been removed for simplicity (The complete results are in Supplementary file 1). Black outlines indicate nodes that were significantly impacted in both CD and UC samples; blue outlines indicate nodes that were significantly impacted in CD samples; and purple outlines indicate nodes that were significantly impacted only in UC samples. The directionalities are shown as yellow or turquoise bars for inferred upregulation or downregulation, respectively. The order is as follows: 1) CD and 2) UC. Abbreviations: act, protein activity; p, protein abundance; bp, biological process. HGNC is the namespace for human proteins; GOBP is the namespace for biological processes; MESHD is the namespace for diseases; and GOCC is the namespace for complexes. SFAM and PMIBP are custom namespaces for protein families and biological processes, respectively. The figure was created using Cytoscape v3.7.1 (https://cytoscape.org). The Cytoscape graph was then exported to pdf and the layout was further modified for better readability in Adobe Illustrator 2019 (https://adobe.com).

Figure 4 shows selected signalling pathways that lead to inflammation in the IBD network models. TLR3, 4, and 9 were inferred to be upregulated in both CD and UC colon samples. Accordingly, IFNβ1 was inferred to be upregulated downstream of TLR3 and 9. TLR7, also upstream of IFNβ1 was inferred to be upregulated only in UC. The inferred upregulation of MYD88 (myeloid differentiation primary response gene 88) indicated activation of Th17 and Th1 cells. However, IL6, which is inhibitory to Th1 activation, was also inferred to be upregulated in CD and to a lesser extent in UC. While IRF (interferon regulatory factor) 1 was inferred to be upregulated and IRF8 was inferred to be downregulated in CD colon, their downstream target, the IFNα family, was inferred to be upregulated in both CD and UC colon. CCL2, also known as MCP-1 (monocyte chemoattractant protein-1) and CXCL1 were inferred to be upregulated in CD colon, suggesting increased monocyte and neutrophil chemotaxis, respectively. On the other hand, CXCL8, another stimulant of neutrophil chemotaxis, was inferred to be downregulated in CD colon. The proinflammatory factors AGER (advanced glycosylation end-product specific receptor), KEAP1 (kelch-like ECH-associated protein 1), and IL17A as well as the anti-inflammatory factor NR3C1 (nuclear receptor subfamily 3 group C member 1) were all inferred to be upregulated in CD colon. IRF5, a stimulator of macrophage activation, was inferred to be upregulated only in UC colon. Finally, CHRNA7 (cholinergic receptor nicotinic alpha 7 subunit) was inferred to be downregulated in CD colon.

### Wound healing network model

**Figure 5:**
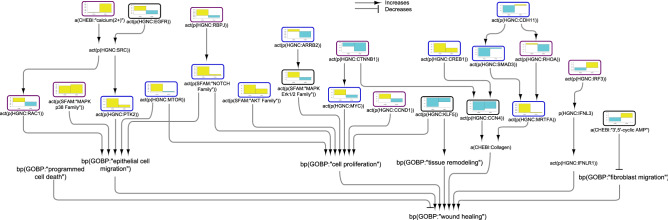
Impact of IBD on signalling leading to wound healing in colonic mucosa. The subgraph extracted from the merged IBD network model shows the iNode enrichment score for each node when the model is scored with comparative transcriptomic data from IBD-affected versus healthy control samples. The values are not comparable between the graphs, and the scale is adjusted on the basis of the highest value for each node. Most intermediate nodes that were not scored have been removed for simplicity (The complete results are in Supplementary file 1). The iNodes leading to “programmed cell death” are shown in Figs. 3 and 6. Black outlines indicate nodes that were significantly impacted in both CD and UC samples; blue outlines indicate nodes that were significantly impacted in CD samples; and purple outlines indicate nodes that were significantly impacted only in UC samples. The directionalities are shown as yellow or turquoise bars for inferred upregulation or downregulation, respectively. The order is as follows: 1) CD and 2) UC. Abbreviations: act, protein activity; p, protein abundance; bp, biological process. HGNC is the namespace for human proteins; GOBP is the namespace for biological processes; MESHD is the namespace for diseases; and GOCC is the namespace for complexes. SFAM and PMIBP are custom namespaces for protein families and biological processes, respectively. The figure was created using Cytoscape v3.7.1 (https://cytoscape.org). The Cytoscape graph was then exported to pdf and the layout was further modified for better readability in Adobe Illustrator 2019 (https://adobe.com).

Finally, we extracted some pathways that lead to wound healing (Fig. 5). Several pathways, including PTK2 (protein tyrosine kinase 2), MTOR (mammalian target of rapamycin), AKT, ARRB2 (arrestin β2)/ERK (extracellular signal-regulated kinase) 1/2, and MYC, which promote would healing, were inferred to be upregulated in CD colon samples. Moreover, while CDH11 and SMAD3 (mothers against decapentaplegic homolog 3) were inferred to be downregulated, the downstream factor MRTFA (myocardin related transcription factor A) was inferred to be upregulated in CD colon. In contrast, CREB1 (cAMP [cyclic adenosine monophosphate] responsive element binding protein 1) was inferred to be upregulated, while CCN4 (cellular communication network factor 4; also known as WISP1 (Wnt family member 1-inducible-signaling pathway protein 1) was inferred to be downregulated, indicating reduced collagen production in CD samples. KLF5, an important regulator of tissue remodelling, was also inferred to be downregulated in CD colon. Interestingly, despite the positive signal from calcium, RAC1 was inferred to be downregulated in UC colon, indicating reduced epithelial cell migration and wound healing. Moreover, MAPK, ERK1/2, CTNNB1 (catenin beta 1)/CCN4, CCND1 (cyclin D1), and KLF5 were also inferred to be downregulated, in alignment with impaired wound healing in UC colon samples. The strong inferred downregulation of RHOA (Ras homolog family member A) was not propagated to significant inhibition of MRTFA in UC colon samples. The inferred upregulation of IRF3 indicated favourable signalling for wound healing. Finally, cAMP, an important blocker of fibroblast migration and, hence, inhibitor of wound healing was inferred to be downregulated in CD and upregulated in UC colon.

### Network model with the nodes that show divergent regulation in CD and UC

**Figure 6:**
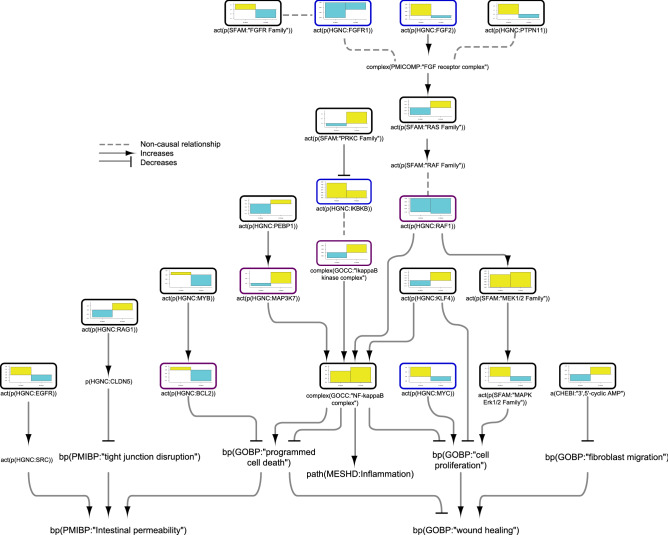
The minimum network with the nodes that show divergent regulation in CD and UC. The subgraph extracted from the merged IBD network model shows the iNode enrichment score for each node when the model is scored with comparative transcriptomic data from IBD-affected versus healthy control samples. The values are not comparable between the graphs, and the scale is adjusted on the basis of the highest value for each node. Most intermediate nodes that were not scored have been removed for simplicity (The complete results are in Supplementary file 1). Black outlines indicate nodes that were significantly impacted in both CD and UC samples; blue outlines indicate nodes that were significantly impacted in CD samples; and purple outlines indicate nodes that were significantly impacted only in UC samples. The directionalities are shown as yellow or turquoise bars for inferred upregulation or downregulation, respectively. The order is as follows: 1) CD and 2) UC. Abbreviations: act, protein activity; p, protein abundance; bp, biological process. HGNC is the namespace for human proteins; GOBP is the namespace for biological processes; MESHD is the namespace for diseases; and GOCC is the namespace for complexes. SFAM and PMIBP are custom namespaces for protein families and biological processes, respectively. The figure was created using Cytoscape v3.7.1 (https://cytoscape.org). The Cytoscape graph was then exported to pdf and the layout was further modified for better readability in Adobe Illustrator 2019 (https://adobe.com).

In summary, while the literature captures causal relationships between the important factors in IBD, scoring of the network model nodes with transcriptomic datasets provides additional information about the complex regulation of signalling pathways in CD and UC in the colon. On the basis of the above analysis, we have extracted a minimum network model that contains the nodes that show divergent regulation in CD and UC (Fig. 6).

The examples above show selected nodes, and an exhaustive list of inferred nodes with their adjusted p values and fold changes are provided in Supplementary file 1.

## Discussion

CD and UC, the main clinical manifestations of IBD, are chronic relapsing inflammatory conditions of the gastrointestinal tract^49^. In this study, we have presented a suite of CBN models that describe relevant molecular processes related to IBD. We assembled BEL-scripted scientific statements derived from original articles into three separate network models that capture the molecular processes involved in intestinal permeability, inflammation, and wound healing responses. As part of network model validation, we scored the network nodes with data on gene expression changes (from colon samples from CD and UC patients) that were expected to trigger perturbation. The scoring also allowed us to investigate the key factors that impact the network and assess the behaviour (activation or inhibition) of molecular entities in the model backbone on the basis of differential gene expression in the selected datasets.

The IBD models highlighted the role of signalling molecules that lead to NF-κB activation. Thus, NOD2, TNF, and several TLR-associated signalling factors (e.g., TLR2, 3, 4, and 9 and the downstream MYD88) were inferred to be upregulated in both CD and UC, thereby confirming that our model captures well-known inflammatory mechanisms in the colon of IBD patients^12,18^. TLR signalling plays a pivotal role in innate immune responses in IBD. A recent study showed that *Tlr4-*, *Tlr2-*, or *Myd88*-knockout mice exposed to dextran sulfate sodium (DSS) show more severe colitis than wildtype mice^50^. These authors also reported that cell proliferation is markedly decreased in *Myd88*^−/−^ and *Tlr4*^−/−^ mice, suggesting that TLR signalling is important for maintaining epithelial barrier function.

PPARG is expressed at high levels in the colon, and the role of this nuclear receptor in controlling inflammation has been well documented in several animal models of colitis and in patients with UC^51^. Several factors have been shown to regulate its activity, including 15‐deoxy‐prostaglandin J2, polyunsaturated fatty acids, and glycerol kinase^51,52^. On the other hand, STAT5B has been shown to inhibit PPARG transcriptional activity through inhibition of AF-1 (ligand-independent activation function region-1)^53^. Accordingly, in our model, the upstream regulator STAT5B was inferred to be upregulated, while PPARG was inferred to be downregulated in CD and UC.

Some studies have shown that NLRP3 inflammasome-induced IL1β aggravates colitis, and inhibition of NLRP3 inflammasome activity has been proposed as a therapeutic strategy for treatment of colitis^23,54,55^. In our model, factors upstream of inflammasomes and IL1β activation, namely the G protein-coupled receptor FFAR2 and PYCARD, were upregulated, although this upregulation reached statistical significance only in UC colon samples. Strikingly, NLRP3 activity was reduced in both UC and CD samples, although IL1β activity was significantly increased in UC patients. These results highlight the important contribution of inflammasome sensors other than NLRP3 in the activation of IL1β in UC. Animal experiments have also implicated the inflammasome sensors NLRP6^56^, NLRC4^57^, and AIM2^58^ in the exacerbation of colitis. Nonetheless, the role of inflammasomes is still controversial, with reports indicating their protective and detrimental effects on colitis^59^. In this regard, it has been reported that NLRP3 inflammasome-deficient mice show exacerbation of DSS- and 2,4,6-trinitrobenzenesulfonic acid (TNBS)-induced colitis and reduced production of IL1β^60,61^. Similarly, NLRP3 is protective in the oxazolone model of colitis, and disease severity can be ameliorated by exogenous administration of IL1β or IL18^62^. The reduced activity inferred in UC samples in the present study might lead to lower IL1β activity than required as well as reduced inflammasome-independent effects for NLRP3^54,63,64^ and may, therefore, account for the development of UC.

The NF-kB upstream regulator IκB kinase (IKK) complex and TNF-mediated NF-κB were inferred to be significantly activated only in UC, with IKK showing reduced activation in CD samples. Moreover, the positive NF-κB activators KLF4 (Krüppel-like factor 4) and SYK^65,66^ were also downregulated in CD samples, although the downregulation reached significance only for KLF4; this causal inconsistency evidences alternative upstream mechanisms for NF-κB activation in CD patients and highlights the complex role of NF-κB in inflammation. Accordingly, besides their well-established pro-inflammatory functions, NF-κB and IKK play an essential role in initiating the resolution of inflammation, as reported previously^67,68^. Consequently, our model may reflect divergent pro-inflammation and pro-resolution programs in UC and CD^69^.

Our IBD models also showed a causal inconsistency in the regulation of RAF1. Thus, the RAS family node, the downstream target of the FGF receptor complex, was inferred to be downregulated in CD and upregulated in UC. Nonetheless, the downstream prosurvival and antiapoptotic RAF1^70^ was inferred to be significantly downregulated in UC samples, pointing to enhanced apoptotic programs in UC. Accordingly, RAF1 has been shown to induce the survival of colonic epithelium through activation of NF-κB in a DSS mouse model of UC^71^. Notably, in the present study, we also found a causal inconsistency between the reduction of RAF1 activity and the increased activity of MEK (mitogen-activated protein kinase kinase) 1/2, suggesting alternative upstream inducers of MEK1/2. In this regard, the activation of BRAF, a proto-oncogene strongly linked to CD-associated colorectal cancer, has been shown to override RAF1 signalling and activate MEK1/2^72,73^.

UC is mostly associated with a Th2 immune response because of the increased intestinal expression of the Th2-associated cytokines IL5, IL13, and IL4^74,75^. Strikingly, in the present study, IL13 was inferred to be upregulated only in CD, while IL4 was inferred to be downregulated only in UC colon. Anti-inflammatory cytokines, such as IL4, IL10, and, partly, IL13, exert an immunosuppressive effect in the colon^76^ by decreasing the production of pro-inflammatory cytokines such as TNF and IL1^77^. IL13 also drives the differentiation of M1 macrophages toward the pro-resolving M2 macrophage phenotype^78^. Consequently, the reduction of IL4-mediated immunomodulatory effects and the lack of significant IL13 activation inferred in UC samples appear to have important implications for the pathogenesis of the disease^79^. Further to this, the inflammation network model illustrates some intriguing differences in the mechanisms underlying UC and CD. The pro-inflammatory factors AGER, KEAP1, and IL17A as well as the anti-inflammatory factor NR3C1 were all inferred to be significantly upregulated in CD colon. On the other hand, IRF5 (interferon regulatory factor 5), a stimulator of macrophage activation, was inferred to be upregulated only in UC colon, evidencing that CD and UC represent different diseases not only macroscopically but also at a molecular level^68^.

Finally, the activity of CHRNA7 was inferred to be significantly downregulated only in CD colon. Several studies have shown that the CHRNA7 agonist nicotine ameliorates colitis in the DSS mouse model of colitis^80^ and in clinical trials^81^. The CHRNA7 agonist anabasine also reduces TNF expression and improves TNBS-induced colitis^82^. These data highlight that disruption of nicotinic cholinergic systems play an important role in the aetiology of IBD^82^ and that engagement of CHRNA7 might have a protective effect in CD.

The loss of epithelial barrier function observed in IBD is attributed to enhanced apoptosis and/or alterations in the expression and architecture of junctional complexes. This is clearly reflected by the intestinal permeability network model, which implied that intrinsic and extrinsic apoptosis pathways—exemplified by CASP8 and TP53, respectively—are promoted in UC samples but not in CD. On the other hand, the antiapoptotic MYB/BCL2 activity was inferred to be significantly decreased in UC and increased in CD, indicating deficient regulatory mechanisms of apoptosis in UC. Accordingly, previous studies point to the fact that colonic epithelium from patients with UC presents higher rates of apoptosis than controls, while the colonic epithelium of patients with CD does not present any difference^83^. MAPK8/JNK1 and calcium, a known activator of JNK, were inferred to be upregulated in UC colon and are linked to tight junction disruption. Moreover, MAP2K7 was also significantly activated in UC but not in CD samples; together with calcium-associated SRC activation, MAP2K7 has been shown to trigger the translocation of OCLN and TJP1 from tight junctions to extracellular spaces, further linking UC with tight junction disruption. Conversely, AHR, which has been linked to increased TJP1, TJP3, and CLDN4 expression levels, was inferred to be upregulated in CD colon, indicating a protective effect on barrier function. The inferred upregulation of NOTCH in CD samples further indicates an increased barrier function in CD colon. However, the barrier promoting factor RAG1^84^ and the adherent junction protein CDH1/E-cadherin, were also inferred to be downregulated in CD colon. These data exemplify the fine-tuned balance between contrasting processes that regulate the integrity of the intestinal mucosal barrier and how their dysregulation can lead to IBD.

The wound healing model evidenced divergent gene regulation in CD and UC samples. Thus, several factors involved in cell proliferation and subsequent wound healing, including mTOR, AKT, ARRB2/ERK1/2, and MYC, were inferred to be significantly upregulated in CD but not in UC colon samples. Moreover, ERK1/2 was significantly downregulated in UC. With regard to ARRB2, insulin-like growth factor-1 has been demonstrated to contribute to mucosal repair through an ARRB2-mediated ERK1/2 mechanism in DSS-induced colitis^85^. These results suggest that proliferation is promoted in CD to compensate for the tissue damage accompanying the disease, while, in UC, lack of cell proliferation may indicate a cause for the onset of inflammation. Along these lines, CTNNB1/CCN4, CCND1, RAC1, and KLF5, which promote wound healing responses, were also inferred to be downregulated in UC colon samples. Moreover, cAMP, an inhibitor of fibroblast migration and wound healing, was inferred to be downregulated in CD and upregulated in UC colon. In sharp contrast, MAPK p38, which is associated with epithelial cell migration and wound healing, was significantly upregulated in UC samples, reflecting the complex regulation of wound healing responses in IBD. A causal inconsistency was observed in the CDH11/SMAD3 axis, which was inferred to be significantly downregulated, while the downstream MRTFA was inferred to be upregulated in CD colon. Of note, the strong inferred downregulation of RHOA in UC did not lead to significant inhibition of MRTFA. In agreement with the promotion of wound healing-associated pathways in CD when compared with UC, MRTFA has been linked to collagen production and wound healing^86^, further supporting the pro-resolution programs in CD when compared with UC. Conversely, CREB1 was inferred to be upregulated in CD, while the downstream CCN4/WISP1—which is liked to fibroblast migration and proliferation as well as collagen production^87^—was inferred to be downregulated in both UC and CD, indicating defective wound healing responses.

Health and disease emerge as a self-organized order from the complex interactions among several biological components. Through the development of computational models that integrate data from clinical and fundamental research, systems medicine has the potential to clarify how these interactions form functional networks and how deregulation of these networks underlies complex diseases such as IBD. Thus, the comprehensive approach of systems medicine can help identify disease biomarkers and drug targets as well as develop novel therapeutic strategies, thereby driving translational advances. Current healthcare practices focus on treating a disease after the onset of illness. Systems medicine has enabled a transition from this reductionist approach to P4 medicine, a new comprehensive and predictive paradigm in medicine for predictive, preventive, personalized, and participatory healthcare. Moreover, with the computational integration of personalized data, including genomic, epigenetic, environmental, and medical data, systems medicine enables the personalization of diagnosis, prognosis, and treatment decisions to address the complexity of human diseases.

In conclusion, this representation of barrier defence, inflammation, and wound healing processes through CBN models constitutes a powerful resource for systems medicine^41^. It is increasingly recognized that CD and UC represent different molecular diseases^88^. Our IBD network model highlights the divergent molecular mechanisms underlying both pathologies, with some of the differences seemingly being a result of Th1- and Th2-mediated responses in CD and UC, respectively. A greater number of divergent factors and pathways between CD and UC appear in the barrier function and wound healing models, suggesting that the differences between both pathologies may lie, at least in part, in a more pronounced barrier dysfunction and/or in defective mechanisms in the resolution phase of inflammation in the colon of UC patients when compared with CD patients. Our model demonstrates causal inconsistencies in several signalling pathways. This might be explained by the contribution of additional undefined incoming signals to the reported pathways. Currently, only 150 out of 320 activity nodes in the model are iNodes. When more nodes can be scored, we may find explanations to the causal inconsistencies in the affected signalling pathways. Additionally, our understanding of these diseases comes mainly from studies performed in animal and in vitro models, which not always translate to human disease. In fact, our data-driven approach revealed significant gaps in existing knowledge on IBD. Of the 355 iNodes that were inferred to be significantly regulated in human CD or UC, only 106 currently exist in the model. Future work will entail investigation of these molecules and additional players in IBD pathology.

Our work demonstrates that IBD network models are a valuable tool for scoring high-throughput data to gain molecular mechanistic insights into the development of the disease. Together with gene expression data from well-controlled clinical studies, the IBD network model suite can help identify the mode of action of novel therapeutic candidates or predict treatment outcomes. A central challenge of IBD network models will be to apply pharmacogenetics findings to clinical practice through development of gene expression assays for disease monitoring and diagnostics. In summary, the IBD network model suite represents a powerful tool for precision medicine.

## Supplementary Information

Supplementary Information.

## Data Availability

The network models are available for browsing and download in the CBN database (http://causalbionet.com/).
